# The prognostic significance of the advanced lung cancer inflammation index in patients with unresectable metastatic colorectal cancer: a retrospective study

**DOI:** 10.1186/s12885-019-5468-9

**Published:** 2019-03-18

**Authors:** Masatsune Shibutani, Kiyoshi Maeda, Hisashi Nagahara, Tatsunari Fukuoka, Shinji Matsutani, Kenjiro Kimura, Ryosuke Amano, Kosei Hirakawa, Masaichi Ohira

**Affiliations:** Department of Surgical Oncology, Osaka City University Graduate School of Medicine, 1–4–3 Asahi-machi Abeno–ku, Osaka City, Osaka Prefecture 545-8585 Japan

**Keywords:** Metastatic colorectal cancer, Prognosis, Inflammatory marker, Body mass index

## Abstract

**Background:**

Growing evidence indicates that inflammation contributes to cancer progression, and several inflammatory markers have been reported to be associated with the clinical outcomes in patients with various types of cancer. Recently, the advanced lung cancer inflammation index (ALI) has been developed as a prognostic marker in patients with lung cancer. The difference between the ALI and the inflammatory markers reported in the previous studies is that the ALI contains not only indices related to inflammation but also the body mass index (BMI), which was reported to correlate with the sarcopenic status. The aim of this study was to evaluate the prognostic significance of the ALI in patients with unresectable metastatic colorectal cancer.

**Methods:**

We retrospectively reviewed a database of 159 patients who underwent combination chemotherapy for unresectable metastatic colorectal cancer between 2008 and 2016. The BMI was calculated by dividing the weight by height squared. The neutrophil-to-lymphocyte ratio (NLR) was calculated from a blood sample by dividing the absolute neutrophil count by the absolute lymphocyte count. The ALI was defined as follows: ALI=BMI × serum albumin concentration/NLR.

**Results:**

The overall survival rate was significantly worse in the low-ALI group than in the high-ALI group (*p* < 0.0001). Furthermore, the ALI was an independent prognostic factor for the overall survival (hazard ratio: 2.773, 95% confidence interval: 1.773–4.335, *p* < 0.001).

**Conclusions:**

A newly developed prognostic marker, the ALI, was found to be a novel prognostic marker in patients with unresectable metastatic colorectal cancer as well as in patients with lung cancer.

## Background

Growing evidence indicates that inflammation contributes to cancer progression [1, 2], and several inflammatory markers, such as the neutrophil-to-lymphocyte ratio (NLR), lymphocyte-to-monocyte ratio (LMR), Glasgow prognostic score (GPS) and the C-reactive protein-to-albumin ratio (CRP/ALB ratio), have been reported to be associated with the clinical outcomes in patients with various types of cancer, including colorectal cancer [3–10].

Recently, the advanced lung cancer inflammation index (ALI), which is based on the body mass index (BMI), serum albumin concentration and NLR, was developed as a prognostic marker in patients with lung cancer [11]. The difference between the ALI and the inflammatory markers reported in previous studies is that the ALI contains not only indices related to inflammation but also the BMI, which has been reported to correlate with the sarcopenic status [12]. Sarcopenia is an important component of cancer cachexia syndrome and a significant prognostic factor in patients with malignant disease [13, 14]. Although there are few patients with cachexia among the patients who undergo curative resection for colorectal cancer, patients suffering from cachexia are present in definite proportions among those with unresectable metastatic colorectal cancer. Therefore, the ALI, which contains the BMI, is thought to be a more accurate prognostic marker than others.

The aim of this study was to evaluate the prognostic significance of the ALI in patients with unresectable metastatic colorectal cancer.

## Methods

Between 2008 and 2016, a total of 159 patients underwent combination chemotherapy for unresectable metastatic colorectal cancer as a first-line treatment at the Department of Surgical Oncology of Osaka City University*.*

Blood samples were obtained and the height and the weight were measured within a period of one week prior to the initiation of chemotherapy. We assayed the serum C-reactive protein (CRP) and albumin concentrations by a chemiluminescent immunoassay (Wako, Osaka, Japan) according to the manufacturer’s protocol. We determined the differential leukocyte count by an XE-5000 hematology analyzer (Sysmex, Kobe, Japan) according to the manufacturer’s protocol. The BMI was calculated by dividing the weight by the height squared (kg/m^2^). The NLR was calculated as absolute neutrophil count divided by absolute lymphocyte count from a complete blood count with differential. The ALI was defined as follows: ALI=BMI × serum albumin concentration/NLR. The modified GPS (mGPS) was defined according to the methods of a previous report [9], using the combination of the serum CRP and albumin levels: patients with a CRP level of < 1.0 mg/dl were allocated a score of 0; those in whom the CRP and albumin levels were ≥ 1.0 mg/dl and ≥ 3.5 g/dl, respectively, were allocated a score of 1; and those in whom the CRP and albumin levels were ≥ 1.0 mg/dl and < 3.5 g/dl, respectively, were allocated a score of 2. Computed tomography (CT) was used to evaluate the skeletal muscle mass. Manual tracing using CT imaging at the level of the third lumbar vertebra was used to measure the cross-sectional areas of the right and left psoas muscle. The psoas muscle mass index (PMI) was calculated as the cross-sectional areas divided by height (cm^2^/m^2^).

Continuous variables were compared using the Mann-Whitney test. Survival curves were made using the Kaplan-Meier method. Patients whose follow-up was discontinued were treated as censored cases in the survival analysis. Differences in the survival curves were assessed using the log-rank test. To evaluate the prognostic factors associated with overall survival, multivariate Cox proportional hazard model was applied. The association between the BMI and the PMI was evaluated by Spearman’s rank correlation coefficient. Statistical analyses were performed using the SPSS software program, version 19.0 (IBM, Armonk, NY, USA). *P* values of < 0.05 were considered to be statistically significant.

This research conformed to the provisions of the Declaration of Helsinki. Written informed consent for the retrospective analysis of the clinical data was obtained from all participants. This retrospective study was approved by our institutional review board (approval No.926).

## Results

### Patient characteristics

The baseline characteristics of the patients are shown in Table 1. Eighty-seven patients were males and 72 patients were females. The median age of the patients was 65 years (range: 18 to 89). According to the definition of the Eastern Cooperative Oncology Group performance status (PS), PS was evaluated as 0 in 139 patients, 1 in 17 patients, and 2 in 3 patients. Thirty-nine patients were right-side colorectal cancer, and 120 were left-side colorectal cancer. One hundred and six patients had single-organ metastasis, and 53 had multiorgan metastases. Oxaliplatin, irinotecan plus 5-fluorouracil/leucovorin, or the prodrug of 5-fluorouracil were administered in all patients as first-line chemotherapy. The treatment regimens, which were considered to have the same efficacy, were used for all patients in the current study [15–17]. Five-fluorouracil+leucovorin+oxaliplatin (FOLFOX) were administered in 74 patients, capecitabine+oxaliplatin (CapeOX) were administered in 52, 5-fluorouracil+leucovorin+irinotecan (FOLFIRI) were administered in 25, and S-1 + oxaliplatin (SOX) were administered in 8. Molecular-targeted drug in combination with cytotoxic drugs was administered in 103 patients. The median duration of follow-up was 21.6 months (range: 1.2 to 94.0 months). Forty-eight patients were alive at the end of the follow-up period, and 111 patients died during the follow-up period.

**Table 1:** Patients’ characteristics

Age (years)	
Median (range)	65 (18–89)
Gender	
Male	87
Female	72
Performance status	
0	139
1	17
2	3
Location of primary tumor	
Right side	39
Left side	120
Histological type	
Well, Moderately	142
Poorly, Mucinous	17
RAS status	
Wild type	63
Mutant type	54
Unknown	42
Detection of unresectable tumor	
Synchronous	106
Metachronous	53
Number of organs affected by metastasis	
One organ	106
Multiple organs	53
Peritoneal dissemination	
Negative	124
Positive	35
First-line chemotherapy regimen	
FOLFOX	74
CapeOX	52
FOLFIRI	25
SOX	8
Molecular-targeted therapy	
Bevacizumab	85
Cetuximab	11
Panitumumab	7
None	56
BMI (kg/m^2^)	
Median (range)	21.71 (15.30–33.76)
Serum albumin concentration (g/dl)	
Median (range)	3.9 (2.5–4.9)
NLR	
Median (range)	2.41 (0.58–13.56)
ALI	
Median (range)	37.92 (4.61–175.07)

*FOLFOX* 5-fluorouracil+leucovorin+oxaliplatin, *CapeOX* Capecitabine+oxaliplatin, *FOLFIRI* 5-fluorouracil+leucovorin+irinotecan, *SOX* S-1 + oxaliplatin, *NLR* Neutrophil-to-lymphocyte ratio, *ALI* Advanced lung cancer inflammation index, *BMI* Body mass index

### Relationships between the ALI (and its components) and clinicopathological factors

Table 2 shows the relationship between the ALI (and its components) and the clinicopathological factors. The ALI had no significant relationship with any of the clinicopathological factors, with the exception of the timing of the detection of metastatic tumors.

**Table 2:** The correlations between the ALI (and its components) and the clinicopathological factors

	ALI		BMI (kg/m^2^)		Albumin (g/dl)		NLR	
	Median (range)	*p*-value	Median (range)	*p*-value	Median (range)	*p*-value	Median (range)	*p*-value
Age								
< 65	34.17 (4.68–174.81)		21.73 (15.48–29.91)		4.0 (2.7–4.9)		2.66 (0.71–12.57)	
≥ 65	38.52 (4.61–175.07)	0.367	21.50 (15.30–33.76)	0.683	3.8 (2.5–4.8)	0.023	2.18 (0.58–13.56)	0.112
Gender								
Male	38.42 (4.61–174.81)		21.80 (15.32–29.91)		3.9 (2.5–4.7)		2.44 (0.71–13.56)	
Female	36.81 (7.41–175.07)	0.341	20.88 (15.30–33.76)	0.246	3.9 (2.9–4.9)	0.714	2.41 (0.58–8.85)	0.459
Location of primary tumor								
Right side	41.44 (4.61–175.07)		22.05 (15.32–33.76)		3.9 (2.7–4.8)		1.95 (0.58–13.56)	
Left side	33.70 (7.41–109.73)	0.137	21.39 (15.30–29.91)	0.628	3.95 (2.5–4.9)	0.617	2.53 (0.94–8.85)	0.069
Histological type								
Well, Moderately	36.81 (4.61–175.07)		21.50 (15.30–33.76)		3.9 (2.5–4.9)		2.48 (0.58–13.56)	
Poorly, Mucinous	39.39 (12.96–101.80)	0.442	21.79 (16.98–27.34)	0.962	3.9 (3.2–4.6)	0.786	2.03 (1.05–4.95)	0.375
RAS status								
Wild type	40.35 (4.61–109.73)		21.20 (15.30–33.76)		4.1 (2.5–4.8)		2.08 (0.94–13.56)	
Mutant type	37.29 (4.68–175.07)	0.203	21.80 (16.00–29.91)	0.652	4.0 (2.7–4.9)	0.829	2.66 (0.58–12.57)	0.075
Detection of unresectable tumor								
Synchronous	28.98 (4.61–175.07)		21.49 (16.00–33.76)		3.85 (2.5–4.8)		2.69 (0.58–13.56)	
Metachronous	40.43 (9.31–109.73)	0.016	22.02 (15.30–29.29)	0.680	4.0 (3.1–4.9)	0.020	2.08 (0.97–6.33)	0.028
The number of organs affected by metastasis								
One organ	38.21 (7.41–175.07)		21.30 (15.30–29.91)		3.9 (2.5–4.9)		2.41 (0.58–7.77)	
Multiple organs	36.13 (4.61–174.81)	0.968	21.80 (16.89–33.76)	0.535	3.9 (2.7–4.8)	0.718	2.48 (0.71–13.56)	0.930
Peritoneal dissemination								
Negative	35.42 (7.41–175.07)		21.60 (15.30–33.76)		3.9 (2.5–4.9)		2.52 (0.58–8.85)	
Positive	39.39 (4.61–98.33)	0.365	21.86 (16.98–25.65)	0.339	4.0 (2.7–4.7)	0.190	2.04 (0.94–13.56)	0.282
Molecular targeted therapy								
Without	35.15 (11.17–174.81)		22.44 (16.12–33.76)		3.85 (2.5–4.8)		2.42 (0.71–6.07)	
With	38.42 (4.61–175.07)	0.977	21.00 (15.30–29.91)	0.105	4.0 (2.7–4.9)	0.281	2.41 (0.58–13.56)	0.986

*ALI* Advanced lung cancer inflammation index, *BMI* Body mass index, *NLR* Neutrophil to lymphocyte ratio

### Classification according to the ALI

**Fig. 1:**
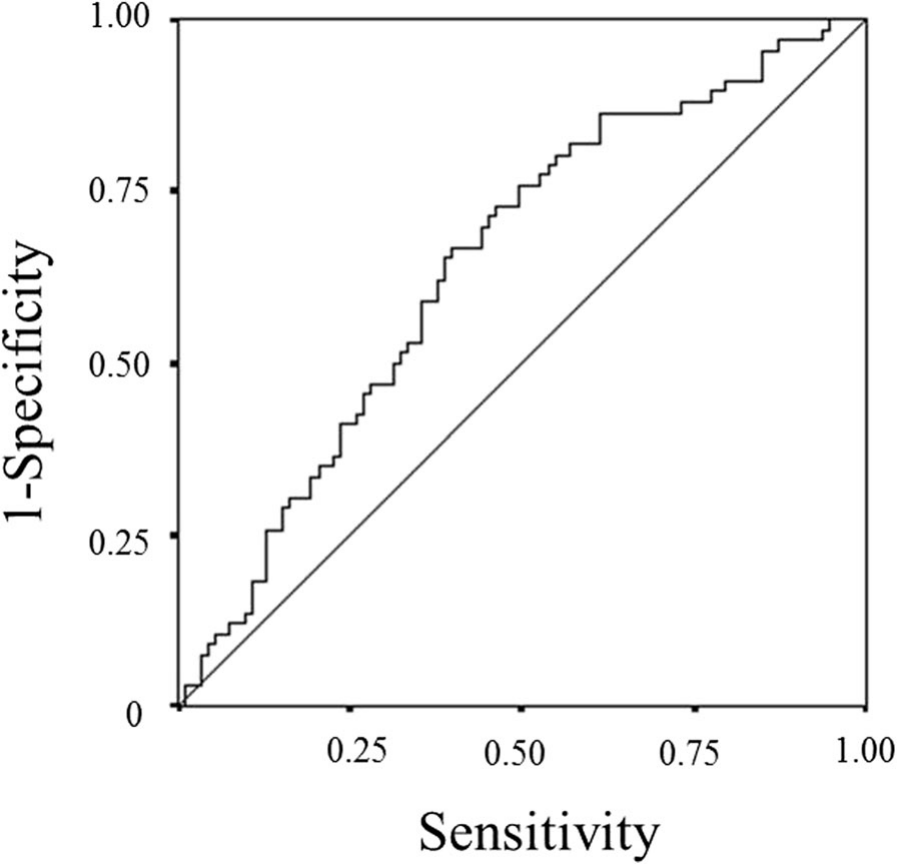
A receiver operating characteristic curve analysis of the advanced lung cancer inflammation index (ALI) in patients with unresectable metastatic colorectal cancer. Area under the curve = 0.654, 95% confidence interval = 0.559–0.731, *p* = 0.002

The ALI, which is a continuous variable, was used as the test variable, and the 24.4-month survival (median survival time: 24.4 months) was used as the state variable. We set 28.9 (sensitivity: 72.7%; specificity: 53.8%) as the cut-off value for the ALI by calculating the receiver operating characteristic (ROC) curve (Fig.1). The patients were classified into high-ALI (*n* = 92) and low-ALI (*n* = 67) groups.

### Survival analyses according to the ALI

**Fig. 2:**
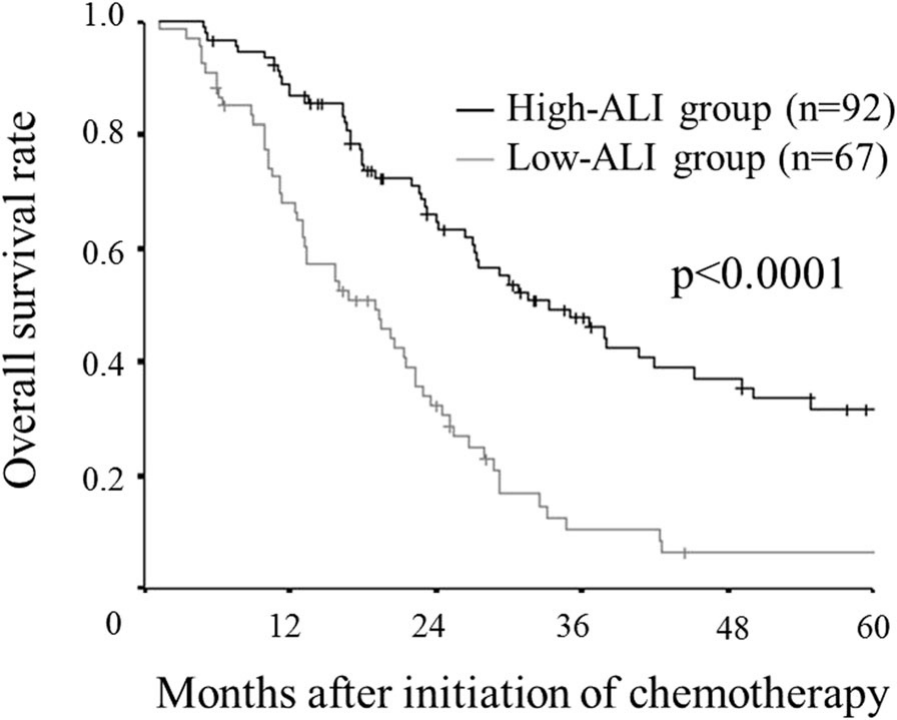
Kaplan–Meier survival curves for the overall survival according to the advanced lung cancer inflammation index (ALI). A low ALI has a detrimental effect on the overall survival (*p* < 0.0001)

The overall survival rate for the low-ALI group was significantly lower than that for the high-ALI group (*p* < 0.0001) (Fig. 2).

### Prognostic factors influencing the overall survival

The correlations between the overall survival and the various clinicopathological factors are shown in Table 3. The univariate analysis showed that the overall survival was significantly associated with the performance status (*p* = 0.021), the location of the primary tumor (*p* = 0.002), the RAS status (*p* = 0.018) and the ALI (*p* < 0.001). A multivariate analysis of these significant variables indicated that the location of the primary tumor (hazard ratio: 2.441, 95% confidence interval: 1.427–4.175, *p* = 0.001) and the ALI (hazard ratio: 2.773, 95% confidence interval: 1.773–4.335, p < 0.001) were independently associated with the overall survaival.

**Table 3:** The correlations between the overall survival and various clinicopathological factors

	Univariate analysis			Multivariate analysis		
	Hazard ratio	95% CI	*p*-value	Hazard ratio	95% CI	*p*-value
Age (≥65 vs. < 65 years)	1.266	0.869–1.845	0.219			
Gender (Female vs. Male)	1.300	0.895–1.888	0.169			
Performance status (≥1 vs. 0)	1.847	1.098–3.106	0.021	1.501	0.767–2.936	0.236
Location of primary tumor (Right side vs. Left side)	1.985	1.273–3.094	0.002	2.441	1.427–4.175	0.001
Histological type (Poorly, Mucinous vs. Well, Moderately)	0.710	0.368–1.370	0.307			
RAS status (Wild type vs. Mutant type)	1.697	1.095–2.628	0.018	1.416	0.902–2.222	0.131
Detection of unresectable tumor (Synchronous vs. Metachronous)	1.087	0.723–1.635	0.687			
Number of organs affected by metastasis (≥2 vs. 1)	1.142	0.770–1.693	0.509			
Peritoneal dissemination (Positive vs. Negative)	1.156	0.732–1.824	0.534			
Molecular-targeted therapy (Absent vs. Present)	1.148	0.783–1.684	0.480			
ALI (Low vs. High)	2.571	1.754–3.769	< 0.001	2.773	1.773–4.335	< 0.001

*CI* Confidence interval, *ALI* Advanced lung cancer inflammation index

### Survival analysis according to the mGPS

**Fig. 3:**
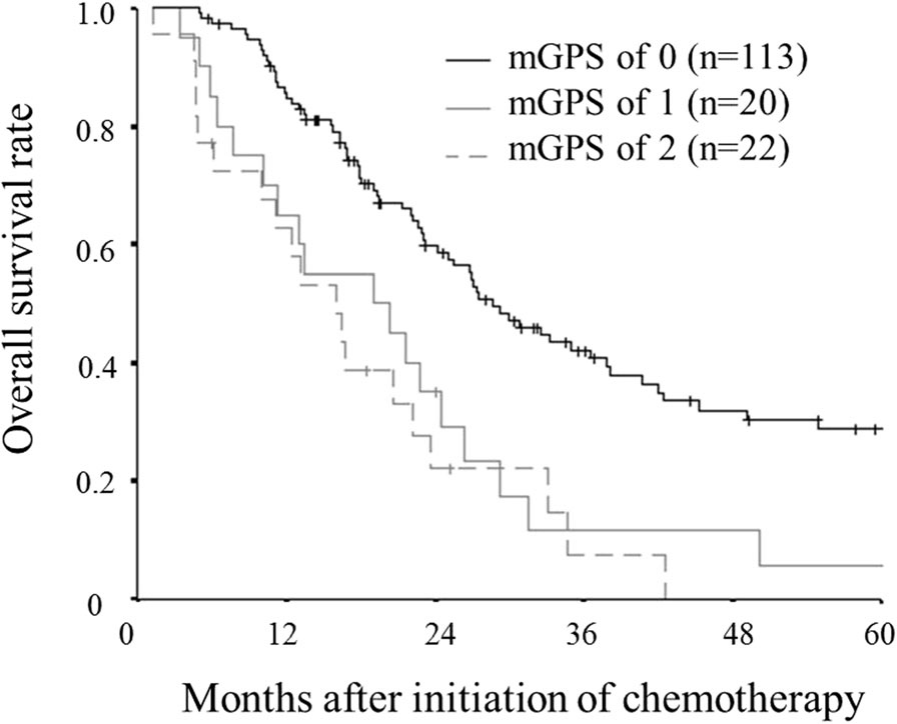
Kaplan–Meier survival curves for overall survival according to the modified Glasgow prognostic score (mGPS). Although there was no significant difference in the overall survival between patients with an mGPS of 1 and those with an mGPS of 2, the overall survival rate of patients with an mGPS of 0 was significantly better than that of those with an mGPS of 1 or 2

Although there was no significant difference in the overall survival between the patients with an mGPS of 1 and those with an mGPS of 2, the overall survival rate of patients with an mGPS of 0 was significantly better than that of those with an mGPS of 1 or 2 (Fig.3).

### Classification according to the BMI

**Fig. 4:**
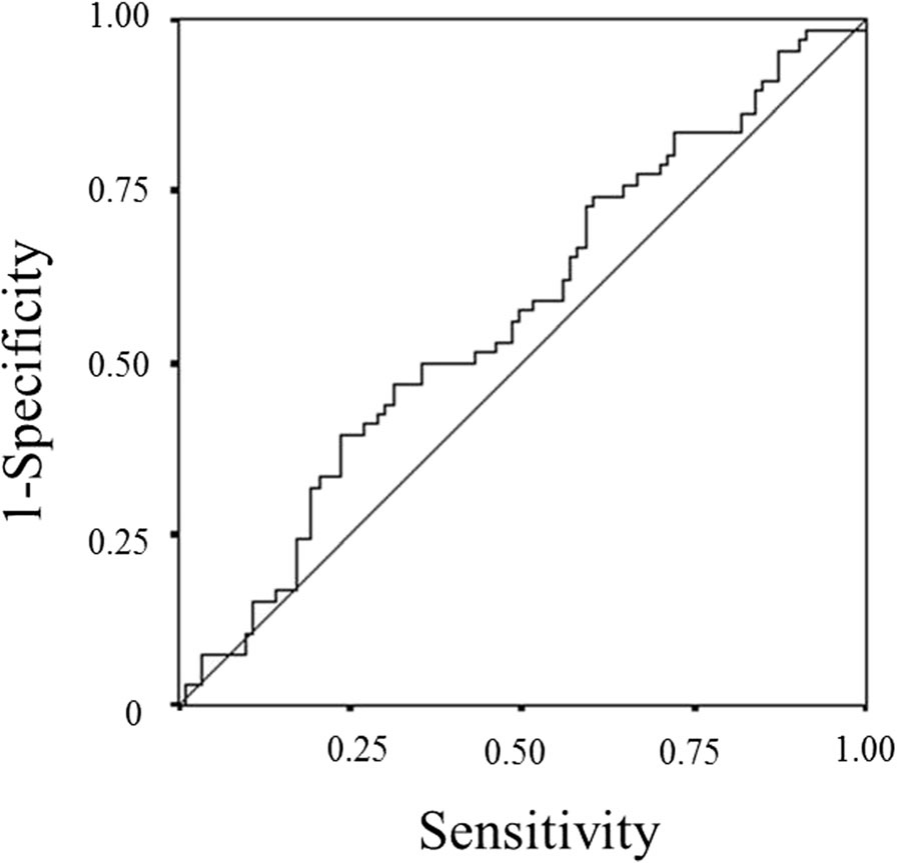
A receiver operating characteristic curve analysis of the body mass index (BMI) in patients with unresectable metastatic colorectal cancer. Area under the curve = 0.570, 95% confidence interval = 0.480–0.660, *p* = 0.133

The BMI, which is a continuous variable, was used as the test variable, and the 24.4-month survival (median survival time: 24.4 months) was used as the state variable. We set 22.66 (sensitivity: 47.0%; specificity: 68.8%) as the cut-off value for the BMI by calculating the ROC curve (Fig.4). The patients were classified into high-BMI (*n* = 60) and low-BMI (*n* = 99) groups.

### Survival analysis according to the BMI

**Fig. 5:**
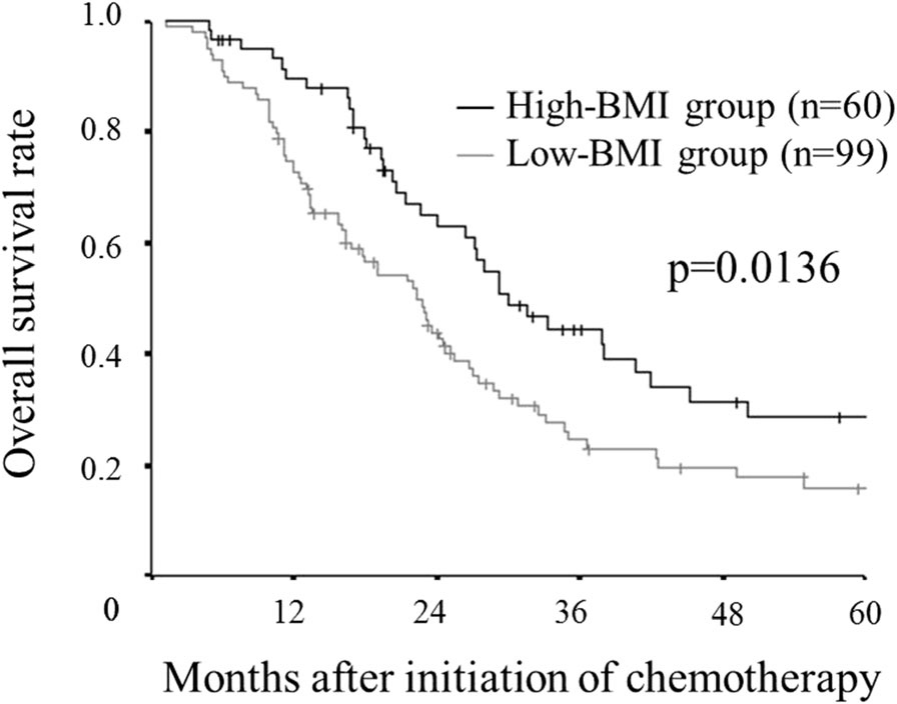
Kaplan–Meier survival curves for the overall survival according to the body mass index (BMI). A low BMI has a detrimental effect on the overall survival (*p* = 0.0136)

**Fig. 6:**
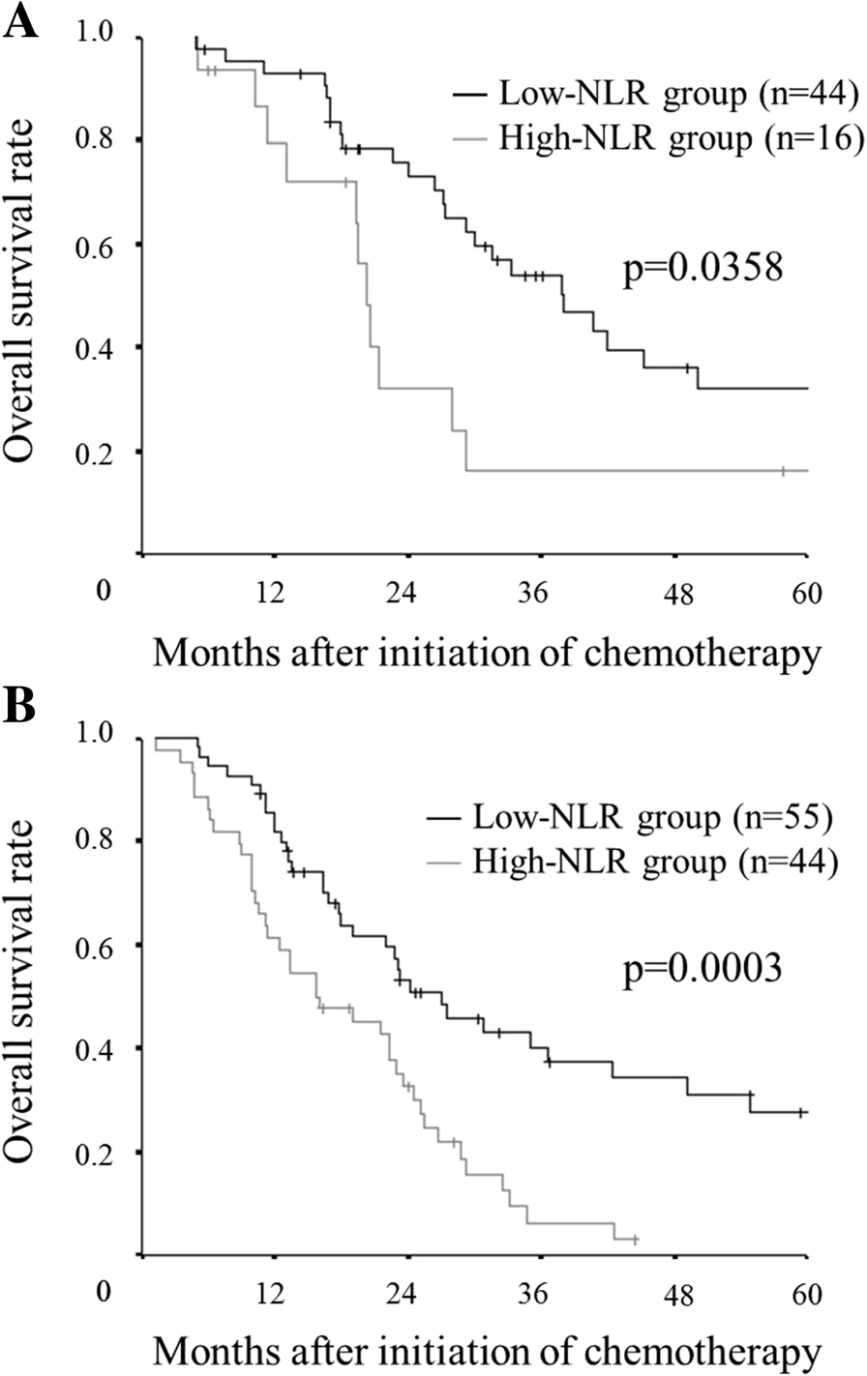
Kaplan–Meier survival curves for overall survival according to the combination of the body mass index (BMI) and the neutrophil to lymphocyte ratio (NLR). In the high BMI group, the overall survival rate in the high NLR group was significantly worse than that in the low NLR group (*p* = 0.0358) (**a**) Similarly, in the low BMI group, the overall survival rate in the high NLR group was significantly worse than that in the low NLR group (*p* = 0.0003) (**b**)

The overall survival rate for the low-BMI group was significantly lower than that for the high-BMI group (*p* = 0.0136) (Fig. 5). Furthermore, we performed a survival analysis according to the combination of the BMI and the NLR. We set 3.0 as the cut-off NLR according to a previous report [18]. In the high BMI group, the overall survival rate of the high NLR group was significantly worse than that of the low NLR group (*p* = 0.0358). Similarly, in the low BMI group, the overall survival rate of the high NLR group was significantly worse than that of the low NLR group (*p* = 0.0003) (Fig.6). The combination of the BMI and the NLR had greater prognostic value than the BMI alone.

### Relationship between the BMI and the PMI

**Fig. 7:**
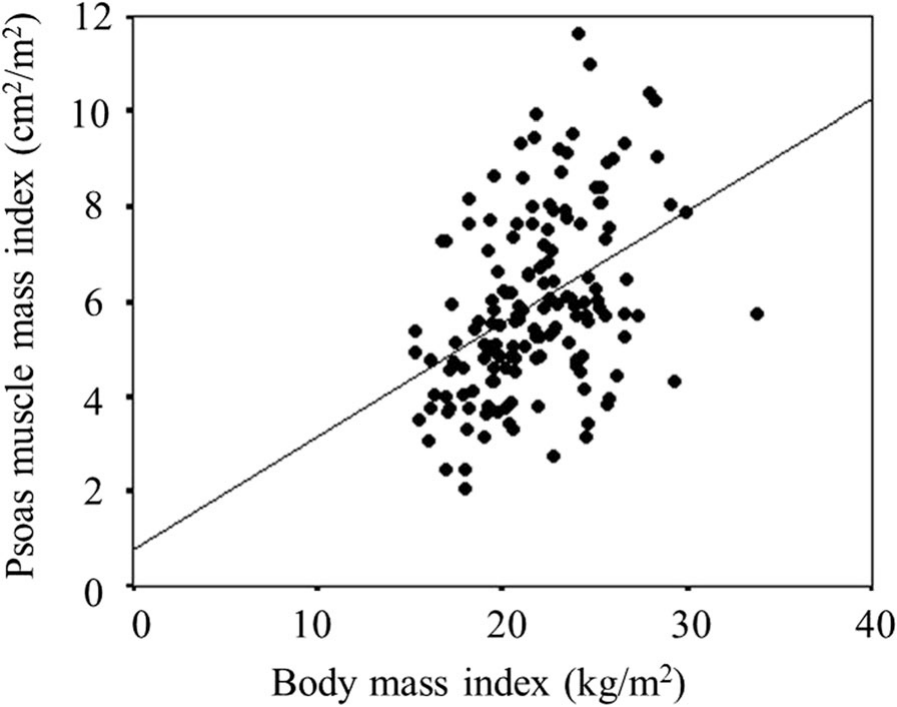
The correlation between the body mass index (BMI) and the psoas muscle mass index (PMI). The BMI was significantly associated with the PMI (r = 0.437, *p* < 0.001)

The BMI was significantly associated with the PMI (r = 0.437, *p* < 0.001) (Fig.7).

### Relationship between the ALI and tolerance of chemotherapy

There was no significant relationship between the ALI and the discontinuation of chemotherapy due to the Grade ≥ 3 adverse events (Table 4).

**Table 4:** The relationships between the ALI and discontinuation due to adverse events

	Without (*n* = 124)	With (*n* = 35)	*P*-value
ALI			
Median (range)	38.06 (4.68–174.81)	31.45 (4.61–175.07)	0.188

*ALI* Advanced lung cancer inflammation index

## Discussion

In this study, we investigated the prognostic significance of the ALI as a marker for predicting the prognosis in patients with unresectable metastatic colorectal cancer. In previous studies, the ALI has only been validated for its usefulness in patients with lung cancer. To our knowledge, this is the first study to assess the prognostic significance of the ALI in patients with colorectal cancer.

Inflammation is known to play an important role in cancer progression, and there have been a lot of reports regarding the utility of the inflammatory markers as a prognostic marker for cancer patients. Systemic inflammation in cancer patients is caused by several mechanisms, such as tissue inflammation induced by tumor growth or invasion, necrosis and local tissue damage and the production of inflammatory mediators induced by the cancer itself and leukocytes [1, 19, 20]. Because systemic inflammation is responsible for cancer growth, invasion, metastasis and resistance to chemotherapy [1, 19, 21], the measurement of the inflammatory markers has prognostic value in cancer patients.

The BMI has been reported to be associated with the presence of sarcopenia, which is an important component of cancer cachexia syndrome and a significant prognostic factor in cancer patients [12–14]. In this study, a significant correlation was found between the BMI and the PMI, which was reported to be correlated with the skeletal muscle mass of the whole body [22] and which is one of the indicators of sarcopenia. However, the BMI cannot accurately reflect the body composition, such as body fat and skeletal muscle, which are necessary for evaluating sarcopenia. There are some patients with sarcopenic obesity who have heavy weight but low muscle mass. Therefore, Kim et al. developed the modified-ALI, based on the quantitative assessment of skeletal muscle, for use instead of the BMI [23]. Nevertheless, the modified-ALI had no additional prognostic value beyond the original ALI [23]. Thus, Kim et al. concluded that the original-ALI, which was easy to calculate, was sufficient for predicting the prognosis in clinical practice. In the present study, even the BMI alone was associated with survival. However, the combination of the BMI and the NLR had greater prognostic value. As cachexia is a consequence of chronic systemic inflammation, the combination of the BMI and inflammatory markers enables a more accurate assessment of cachexia. Thus, the ALI, which includes the BMI and inflammatory markers, has excellent prognostic value.

In addition to the ALI, various inflammatory markers have been reported to be correlated with the prognosis of patients with various types of malignancies. In this study as well as the previous reports, the mGPS was found to be correlated with the prognosis of patients with unresectable metastatic colorectal cancer, and there is no doubt that the mGPS is a useful prognostic marker. However, as mentioned in past reports, when the mGPS is used, many cases are classified into the group with an mGPS of 0, which is considered to have a good prognosis [9]. Even in this study, which contained advanced cases in which the degree of systemic inflammation was likely to be high due to an increased tumor burden, more than 70% of cases were classified into the group with an mGPS of 0. Although both the ALI and the mGPS are useful prognostic markers, there are still some subjects to be considered. Thus, it is difficult to decide which inflammatory marker is superior at the present time.

Although we set 28.9 as the cut-off value of the ALI based on the ROC curve in this study, the range of the cut-off values used in previous reports are relatively broad, with values of 18, 19.5 and 31.1 [11, 23, 24]. The degree of systemic inflammation was reported to be associated with the tumor progression, as with the TNM stage [3]. In addition, even at the same stage, the degree of inflammation may vary depending on the type of cancer. Therefore, the cut-off value of the ALI used in the present study is a provisional value. In this study, an ROC curve analysis, in which the vital status at the median survival was used as the state variable, was performed to determine the optimal cut-off ALI value. Although there were some patients who died within 6 months after the initiation of chemotherapy, the proportion of such patients was only 6.9%, and in many cases long-term follow-up was possible. Thus, the method used in this study to determine the cut-off value was thought to be relatively reliable.

The current study was a retrospective study with a small cohort in single-center. Further prospective studies are required to confirm our findings. In addition, it is necessary to determine the cut-off value for each type of cancer and stage in order to apply the ALI in the clinical setting.

When considering treatment strategies, the contents of treatment should be mainly decided according to the National Comprehensive Cancer Network or European Society for Medical Oncology guidelines, which are based on large-scale clinical data, rather than inflammatory markers such as the ALI. However, the present study—which was based on clinical data—revealed that ALI was a useful preoperative marker in patients with metastatic colorectal cancer and that inflammation was contributed to the cancer development and chemoresistance, both of which are meaningful findings.

## Conclusions

A newly developed prognostic marker, the ALI, which is based on the BMI, serum albumin concentration and NLR, was found to be a novel prognostic marker in patients with unresectable metastatic colorectal cancer as well as in patients with lung cancer. Our findings indicate that the ALI may provide useful information when considering treatment strategies for unresectable metastatic colorectal cancer.

## Abbreviations

ALI: Advanced lung cancer inflammation index
BMI: Body mass index
CapeOX: Capecitabine+oxaliplatin
CI: Confidence interval
CRP/ALB ratio: C-reactive protein to albumin ratio
CT: Computed tomography
FOLFIRI: 5-fluorouracil+leucovorin+irinotecan
FOLFOX: 5-fluorouracil+leucovorin+oxaliplatin
LMR: Lymphocyte to monocyte ratio
mGPS: Modified Glasgow prognostic score
NLR: Neutrophil to lymphocyte ratio
PMI: Psoas muscle mass index
PS: Performance status
ROC curve: Receiver operating characteristic curve
SOX: S-1 + oxaliplatin
